# Diverticular disease: picking pockets and population biobanks

**DOI:** 10.1136/gutjnl-2019-318231

**Published:** 2019-03-02

**Authors:** Rinse K Weersma, Miles Parkes

**Affiliations:** 1 Department of Gastroenterology and Hepatology, University of Groningen and University Medical Center Groningen, Groningen, The Netherlands; 2 Gastroenterology Unit, Addenbrookes Hospital, Cambridge, UK

**Keywords:** diverticular disease, genetics

The last decade has seen an explosion in genome-wide association studies (GWAS) in many diseases. With these—and its worth reminding ourselves that this is the primary goal of GWAS—have come valuable insights into pathogenic pathways. Some have been predictable (eg, variants affecting antigen presentation, co-stimulation and T-cell responses in immune-mediated diseases) and others, unexpected (eg, autophagy in Crohn’s disease). Until recently, such studies were performed in disease cohorts specifically ascertained for the purpose, with clear evidence that the larger the cohort the more loci will be found.^1^

Such disease-specific cohorts with well-defined phenotypes can be assembled where there are engaged clinical communities, but may be tricky to ascertain for more general traits. Into this category falls diverticular disease. Diverticulosis affects up to 50% of the population but was poorly studied at a genetic level until recently, despite clear evidence for its relatively high heritability.^2 3^ In *Gut*, Schafmayer *et al* report on a large-scale analysis using clinical and genetic data from the UK Biobank. This population cohort totals 500 000 individuals, among whom ~32 000 have a recorded diagnosis of diverticular disease according to the international classification of disease (ICD) 9/10 coding. The authors went on to replicate their findings in a hospital-based case–control cohort.^4^ Interestingly, a similar approach was recently deployed by Maguire *et al*, using the same biobank ‘discovery’ dataset and a separate independent hospital-based registry as replication cohort.^5^ The earlier study identified 39 susceptibility loci for diverticular disease and showed that candidate genes reside in plausible biological pathways involved in cell–cell adhesion, membrane transport signalling and intestinal motility.

In this paper by Schafmayer *et al,* 48 susceptibility loci were identified for diverticular disease at a genome-wide significant statistical threshold, with consistent directionality in the discovery and replication cohorts. Out of these, 12 regions were novel compared with the previous publications. GWAS loci usually harbour multiple genes and it is frequently unclear as to which gene is actually causal. The authors, therefore, performed a series of downstream *in silico* analyses to prioritise candidate genes within each locus. To the extent that these approaches have refined the associations seen, the authors highlighted one gene per locus, identified the fact that many of their signals map to introns (non-coding inserts in genes), and analysed layer-specific mRNA expression and fluorescence immunohistochemical staining in colonic biopsies. Of note, it appears that some degree of manual curation was used in the fine mapping routines, and with this the concern that some bias regarding likely causal genes might have bled into the final results. Nevertheless, novel insights into the pathophysiology of diverticular disease are derived, with the suggestion that it is a disorder of intestinal neuromuscular function, mesenteric vascular smooth muscle function and connective fibre support. These, therefore, overlap at least to a significant extent with the conclusions of Maguire *et al*. At this stage, these mechanisms must be viewed as hypotheses to be tested. Confirmation requires more detailed genetic mapping, ascertainment of correlation between the associated genetic variants and gene expression in relevant cell types, and the interrogation of their functional impact.

Now, what to think of the methodology deployed in the current study? Inevitably, there are some trade-offs when using a population cohort as opposed to a clinical cohort, not least in the definition of the disease under study. Schafmayer *et al* used ICD10 coding (K57) to define the case group, which includes both diverticulitis and diverticulosis. Anyone familiar with coding in the clinical setting will be aware of its potential inaccuracies, not least for a condition such as diverticulosis, where many affected individuals are asymptomatic and others may be diagnosed with the condition in the absence of objective evidence and where the true diagnosis is, for example, irritable bowel syndrome. Further, given the high prevalence of diverticular disease in the population, and its increase with age, one might suppose that mis-specification for ‘case’ and ‘control’ status (the latter including many ‘not yet affected’) would be an issue. It seems that Schafmayer *et al* used a rather more inclusive definition than the study of Maguire *et al*, and with the larger sample identified more loci with a genome-wide significance. As has been well recognised previously, in genetic studies of common diseases, cohort size and statistical power really count, even if they come with some loss of phenotyping accuracy.

While the potential diagnostic imprecision inherent in a population database such as the UK Biobank might be viewed as a weakness, the very fact that the cohort is broadly representative of the whole population rather than specifically collected disease cohorts itself provides an important ‘real world’ context for genetic findings. There is a large danger based on the published literature that clinicians view genetic results as more powerful and of greater deterministic value than they actually are. A recent publication highlights this by showing that the penetrance of causal mutations for many monogenic conditions has probably been substantially over-estimated due to its derivation from studies based on tertiary referral cohorts.^6^

The UK Biobank is a prospective cohort study on ~500 000 population-based individuals.^7^ For each participant, a large set of phenotypes (including ICD codes as used in the current study), health-related measurements, diet and lifestyle data, and biomarkers are available. There are ambitious plans to link them to primary healthcare records and prescribing data, as well as possibly collecting stool samples to complement the DNA and data already collected. GWAS has been performed on nearly the whole cohort using an array with more than 750 000 genetic markers. This unprecedented open access database has been made available to the research community, and—given the size of the cohort—represents a rich resource for genetic studies of common diseases. For many gastro-intestinal (GI) disorders, the number of affected individuals within the UK Biobank is substantial. For example, more than 18 000 are recorded to have cholelithiasis, which is more than double the number of individuals included in the largest GWAS meta-analysis on gallstone disease to date.^8^ Similar number of individuals have, for example, gastro-oesophageal reflux disease, colorectal polyps or irritable bowel syndrome. For each of these conditions, there is adequate statistical power to detect genetic associations with high confidence, as nicely shown by the two studies on diverticular disease. Furthermore, other biobanks are also coming on-stream. In the northern part of the Netherlands, ‘Lifelines’, a three generations longitudinal cohort study of 165 000 participants, will have genetic data available soon, allowing for similar analyses or joint analyses with the UK Biobank results.^9^

These resources provide a fantastic chance for researchers to identify genetic risk factors, pathogenetic mechanisms and potential drug targets across the range of GI disorders, as was done for diverticular disease. To date, out of the more than 1000 genetic studies approved by UK Biobank, only a limited number relate to GI disease (see https://www.ukbiobank.ac.uk/approved-research). This is not good enough. Our research community needs to wake up. This is a fantastic opportunity. We should seize it!
